# Idiopathic serontonin syndrome. Can we prevent it?

**DOI:** 10.1192/j.eurpsy.2022.1868

**Published:** 2022-09-01

**Authors:** L. Gallardo Borge, I.D.L.M. Santos Carrasco, P. Marqués Cabezas, L. Rodriguez Andrés, A. Rodriguez Campos

**Affiliations:** 1 Hospital San Pedro, Psychiatry, Logroño, Spain; 2 Hospital Clinico Universitario, Psychiatry, Valladolid, Spain; 3 Hospital Clinico Universitario de Valladolid, Departamento De Psiquiatría, Valladolid, Spain

**Keywords:** Serotonin syndrome, iatrogenic, serotoninergic, polipharmacy

## Abstract

**Introduction:**

Serotonin syndrome is a mild to potentially life-threatening syndrome associated with excessive serotonergic activity within the central nervous system. Serotonin syndrome is associated with medication use, drug interactions and overdose. All drugs that increase central serotonin neurotransmission at postsynaptic 5-HT1A and 5-HT 2A receptors can produce SS.

**Objectives:**

Clinical case and literature review.

**Methods:**

A 74-year-old female, married, diagnosed of major depressive disorder. Treated with: lithium 600 mg, quetiapine 50 mg, venlafaxine 300 mg. The doses had been maintained for the last months. Lithium levels in the normal range.

**Results:**

In an emergency room, she received a tramadol injection because of strong backpain. After a few hours, she felt an overall worsening, sleepiness and lack of response to external stimuli. Given the persistence of the symptoms and decreased appetite along with decreased water intake, she attended to Hospital. She had a high fever, rigidity and myoclonus. Her language was incoherent. Blood tests showed high CK, and high AST and ALT.

**Conclusions:**

SS is a potentially fatal iatrogenic complication of serotonergic polypharmacy. Considered idiopathic in presentation, it appears tipically after initiation or dose escalation of the offending agent to a regimen including other serotonergic agents. While serotonin syndrome is often associated with the use of selective serotonin inhibitors (SSRI), an increasing number of reports are being presented involving the use of tramadol. It is vital that clinicians are aware of the potential for SS when psychotropic and non-psychotropic agents are co-administered to certain patients, such as those with both depression and pain.

**Disclosure:**

No significant relationships.

